# Enhancing Cancer Therapy: Boron-Rich Polyboronate Ester Micelles for Synergistic Boron Neutron Capture Therapy and PD-1/PD-L1 Checkpoint Blockade

**DOI:** 10.34133/bmr.0040

**Published:** 2024-06-26

**Authors:** Yi-Lin Chiu, Wan Yun Fu, Wei-Yuan Huang, Fang-Tzu Hsu, Hsin-Wei Chen, Tzu-Wei Wang, Pei Yuin Keng

**Affiliations:** Department of Material Science and Engineering, National Tsing Hua University, Hsinchu City 300, Taiwan.

## Abstract

Malignant cancers, known for their pronounced heterogeneity, pose substantial challenges to monotherapeutic strategies and contribute to the risk of metastasis. Addressing this, our study explores the synergistic potential of combining boron neutron capture therapy (BNCT) with immune checkpoint blockade to enhance cancer treatment efficacy. We synthesized boron-rich block copolymer micelles as a novel boron drug for BNCT. Characterization was conducted using nuclear magnetic resonance, gel-permeation chromatography, transmission electron microscopy, and dynamic light scattering. These micelles, with an optimal size of 91.3 nm and a polydispersity index of 0.18, are suitable for drug delivery applications. In vitro assessments on B16-F10 melanoma cells showed a 13-fold increase in boron uptake with the micelles compared to borophenyl alanine (BPA), the conventional boron drug for BNCT. This resulted in a substantial increase in BNCT efficacy, reducing cell viability to 77% post-irradiation in micelle-treated cells, in contrast to 90% in BPA-treated cells. In vivo, melanoma-bearing mice treated with these micelles exhibited an 8-fold increase in boron accumulation in tumor tissues versus those treated with BPA, leading to prolonged tumor growth delay (5.4 days with micelles versus 3.3 days with BPA). Moreover, combining BNCT with anti-PD-L1 immunotherapy further extended the tumor growth delay to 6.6 days, and enhanced T-cell infiltration and activation at tumor sites, thereby indicating a boosted immune response. This combination demonstrates a promising approach by enhancing cytotoxic T-cell priming and mitigating the immunosuppressive effects of melanoma tumors.

## Introduction

Boron neutron capture therapy (BNCT) represents a cutting-edge modality in cancer treatment, leveraging the nuclear reaction between boron-10 (^10^B) and thermal neutrons. This method selectively targets tumor cells while sparing adjacent healthy tissue [1]. This approach relies on the preferential accumulation of boron compounds within tumor cells, which, upon irradiation with low-energy neutrons, results in the emission of high-energy alpha particles (^4^He) and lithium-7 nuclei (^7^Li), leading to localized cellular destruction [1]. Numerous studies, encompassing both preclinical and clinical studies, have been conducted to assess the effectiveness and broader applications of BNCT in oncology. Preclinical models have demonstrated BNCT’s capacity for targeted tumor ablation with minimal damage to surrounding healthy tissues, a feature attributed to the selective uptake of boron compounds by tumor cells, thereby facilitating focused radiation therapy and localized tumoricidal effects [2]. In the clinic, BNCT has demonstrated high response rates in patients with head and neck cancers [3], malignant brain tumors [4], and malignant melanomas [5]. Although BNCT holds great promise for cancer treatment, one of the major barriers for BNCT is cancer recurrent in patients, which can be attributed to the non-homogeneous and insufficient loading of the ^10^B drug throughout the solid tumor [6]. Currently, only 2 boron-based drugs are employed in clinical trials: sodium mercaptoundecahydro-closo-dodecaborate (Na_2_B_12_H_11_SH), commonly known as sodium borocaptate (BSH), and borophenyl alanine (BPA), an amino acid derivative of phenylalanine. Nonetheless, neither BSH nor BPA fully meets the criteria for an ideal boron agent in BNCT, underlining the need for continued research and development in this area [7]. Particularly, BPA’s small molecular size leads to unsatisfactory retention within tumor cells and a suboptimal tumor/blood (T/B) ratio, potentially increasing the risk of adverse effects during BNCT [8].

Current research is therefore pivoting toward third-generation boron delivery agents, employing strategies such as encapsulation of boron drug within liposomes, exosomes, polymer micelles, and nanoparticles, or conjugated to peptides, polymers, antibodies, sugars, nucleosides, and porphyrins to improve the specificity and accumulation of the boron agent in tumor cells [1,7]. These novel agents have demonstrated enhanced intracellular uptake, increased boron accumulation, and more precise targeting, resulting in a higher tumor/normal tissue (T/N) ratio and, consequently, greater tumoricidal efficacy compared to BPA. Despite these advancements, the complete eradication of malignant tumors through monotherapy remains challenging due to the inherent heterogeneity of the tumor microenvironment [9] and the ability of cancer cells to evade immune responses by expressing immune checkpoints [10]. To address these hurdles, combination approaches are being explored [11]. Immunotherapy, notably immune checkpoint inhibitors, has achieved remarkable success in certain cancers by reactivating the immune system’s ability to identify and destroy tumor cells [12].

The immunomodulatory effects associated with BNCT treatment have been studied over the past decade [13]. In these studies, the high Linear Energy Transfer (LET) ionizing radiation of BCNT kills the primary tumor and subsequently induces immunogenic cell death (ICD) [1]. ICD then triggers a cytotoxic immune response toward the primary tumor and its metastasis sites. This process involves the release of danger signals such as high mobility group box protein 1, calreticulin, and cytosolic DNA, triggering immunomodulatory responses that include antigen capture, cell migration to lymph nodes, and activation of cytotoxic T cells [14]. Thus, the synergy between BNCT and immunotherapy presents a compelling approach to overcome these challenges, potentially enhancing therapeutic outcomes.

**Fig. 1.**
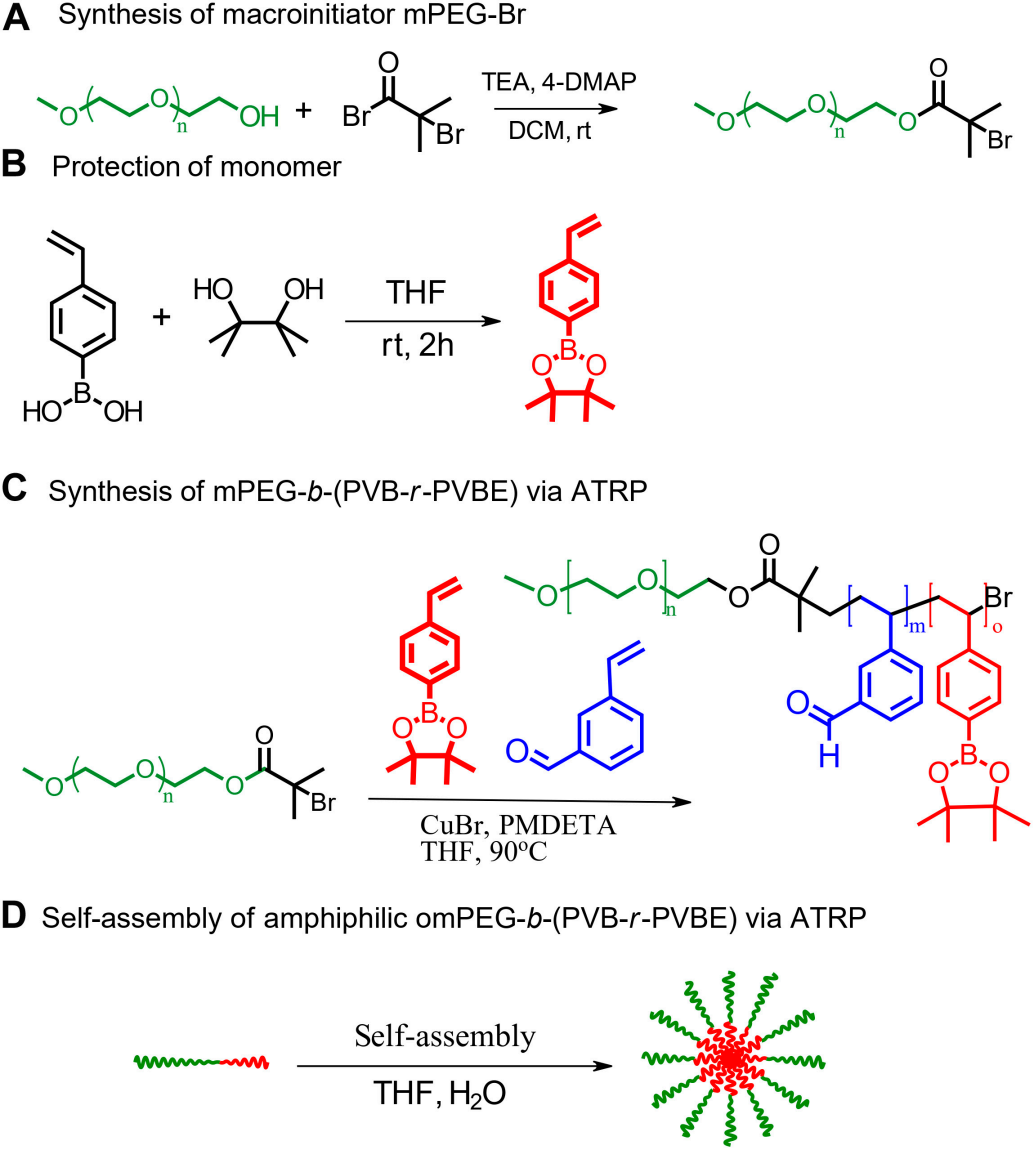
Schematic illustration of the following processes: (A) synthesis of mPEG macroinitiator, (B) protection of 4-vinylphenyl boronic acid using pinacol, (C) preparation of mPEG-*b*-(PVB-r-PVBE) amphiphilic block copolymer through ATRP, and (D) self-assembly of mPEG-*b*-(PVB-r-PVBE) in THF/H_2_O mixture.

To overcome the non-specific delivery and insufficient B-10 distribution in tumor cells, herein, we developed polyboronate ester micelles as a neutron capture agent (Fig. 1). The size of these block copolymer micelles can be tuned by adjusting the structure and length of each block copolymer segment, pH, solvent type, and polymer concentration during the self-assembly processes [15]. Boronic acid-containing copolymers have been widely investigated as stimuli-responsive drug carriers, notably in the formation of core-shell crosslinked micelles and hydrogels. These nanocarrier systems are capable of encapsulating various therapeutics, such as Doxil, cisplatin, and insulin, within the micellar core. These drugs are then released in response to specific stimuli, such as acidic pH or high glucose concentration [16]. In this work, the engineered polyboronate ester micelles are designed to deliver a high payload of B-10 selectively to the tumor microenvironment. Such passive targeted delivery is facilitated through the enhanced permeability and retention (EPR) effect, a phenomenon that allows for the preferential accumulation of nanosized therapeutic agents in tumor tissues due to their unique vascular architecture and microenvironmental characteristics [17].

Using malignant B16-F10 melanoma cells as a model study, the boron-rich polymer micelles showed a 13-fold increase in boron uptake compared to the standard BPA boron drug. In vivo studies on melanoma-bearing BALB-C mice showed an 8-fold increase in boron accumulation in tumor tissues in mice treated with polyboronate ester micelles, as opposed to those treated with BPA. Our study showed that mice treated with the micelles exhibited a tumor growth delay (TGD) of 5.4 days following thermal neutron irradiation, compared to a 3.3-day TGD in mice treated with BPA. We further investigated the effect of BNCT and immune checkpoint blockade combinatorial therapy by administrating murine anti-PD-L1 following BNCT treatment. Mice receiving this combinatorial therapy showed an extended TGD of 6.6 days compared to those treated solely with BNCT. This proof-of-concept study demonstrates that the integration of BNCT utilizing boron-rich polymer micelles with immunotherapy represents a promising strategy for the enhancement of cancer treatment outcomes. This approach synergistically combines the precision of BNCT with the systemic efficacy of immunotherapy, potentially leading to improved therapeutic effectiveness in cancer management.

## Materials and Methods

### Materials and instruments

Poly(ethylene glycol) methyl ether (mPEG_5000_, number average molecular weight [*M*_n_] = 5,000) was purchased from Thermo Scientific. Triethylamine (TEA, 99.5%) and 3-vinylbenzaldehyde (97.0%) were purchased from Sigma-Aldrich. 4-(Dimethylamino)pyridine (DMAP, 98.0%) was purchased from Matrix Scientific. 2-Bromoisobutyryl bromide (BIBB, 97.0%) and copper(I) bromide (CuBr, 98%) were purchased from Alfa Aesar. 4-Vinylphenylboronic acid, pinacol (99%), and *N*,*N*,*N*′,*N*′,*N*′-pentamethyldiethylenetriamine (PMDETA, 98%) were purchased from Acros Organics. Toluene (≥99.7%) was purchased from Sigma-Aldrich. Anhydrous dichloromethane (DCM, 99.5%) and dimethyl sulfoxide (99.5%) were purchased from Avantor. Tetrahydrofuran (THF, AR, 99.5%) was purchased from Duksan. Dialysis membrane (molecular weight cutoff [MWCO] 6 to 8 kDa) was purchased from Biomate. 3-(4,5-dimethylthiazol-2-yl)-5-(3-carboxymethoxyphenyl)-2-(4-sulfophenyl)-2H-tetrazolium (MTS) reagent was purchased from Promega Corporation (CellTiter96 aqueous cell proliferation assay kit). DAPI (4′,6-diamidino-2-phenylindole) and FAST DiI solid; DiIΔ9,12-C18(3), CBS (1,1′-dilinoleyl-3,3,3′,3′-tetramethylindocarbocyanine, 4-Chlorobenzenesulfonate) were purchased from Thermo Fisher Scientific. The FITC anti-mouse CD3 antibody, APC/Cyanine7 anti-mouse CD8a antibody, and PE anti-mouse CD4 antibody were sourced from BioLegend. InVivoMAb anti-mouse PD-L1 (B7H1; Bio X cell), Dulbecco’s Modified Eagle Medium, high glucose (DMEM-HG, Gibco, Thermo Fisher Scientific), and 10% fetal bovine serum (FBS, Gibco, Thermo Fisher Scientific) were stored and used according to the manufacturer’s protocol. The B16-F10 melanoma cells were obtained from the Bioresource Collection and Research Center (BCRC). C57BL/6JNarl male mice (age 4 weeks) were purchased from the National Laboratory Animal Center (Taipei, Taiwan) and were treated according to the National Tsing Hua University of the Institute for Laboratory Animal Research.

The morphology of the polymeric micelle was observed under a 120-kV transmission electron microscope (Hitachi HT7700, Japan). The polymer product was characterized via a VNMRS-700 NMR (nuclear magnetic resonance) spectrometer (Varian, USA) at ambient temperature with tetramethylsilane as an internal standard. The polymer sample was prepared by dissolving with anhydrous DCM and dropping the sample onto the polyethylene (PE) film. Measurement was conducted after the evaporation of the solvent, and the chemical bonding was characterized by Fourier transform infrared spectroscopy (FTIR, Bruker-Vertex 80v-Tensor 27). [18] The polymer micelle size and size distribution were analyzed by the Zetasizer (Malvern Zetasizer ZEN3600). The boron content in cells, organs, and tumor samples was dissolved in nitric acid and hydrofluoric acid, followed by measurement via an inductive coupled plasma mass spectrometer (ICP-MS, Agilent 725). Fluorescent imaging was observed by a laser scanning confocal microscope (ZEISS LSM-780, Germany). The absorbance for ELISA plates and cell viability assays was measured using the Molecular Devices SpectraMax 340PC Microplate Reader. Molecular weights were measured by a gel-permeation chromatography (GPC) system equipped with a Waters 1515 Isocratic HPLC pump, a Waters 2414 refractive index (RI) detector, Waters Styragel columns (HR3, HR4E, 7.8×300 mm), and a Waters temperature control module II thermostat at 40 °C. Lyophilization was carried out in a vacuum freeze-dryer (KINGMECH FD4.5-8P-L-80°C, Taiwan). In vitro and in vivo BNCT experiments were conducted at the Tsing Hua Open-Pool Reactor (THOR, Hsinchu, Taiwan).

### Methods

#### Preparation of poly(ethylene glycol) methyl ether 2-bromoisobutyrate mPEG-Br macroinitiator

The preparation of the mPEG-Br macroinitiator was adapted from the literature [19]. Briefly, the hydroxy end-functionalized methoxy PEG (mPEG-OH) (25.0 g, 5 mmol) was first melted at 60 °C in a round-bottom flask. After cooling to room temperature, 100 ml of anhydrous DCM was added to dissolve the mPEG-OH. Then, TEA (2.8 ml, 20 mmol) and DMAP (2.4 mg, 20 mmol) were added as an acylation catalyst. The mixture was stirred and deoxygenated by bubbling with nitrogen for 30 min. The deoxygenated reaction mixture was then cooled in an ice bath. Then, a solution of BIBB (2.5 ml, 20 mmol) in 10 ml of DCM was added dropwise using a syringe and the reaction was stirred overnight under nitrogen. After the reaction completes, the crude reaction was concentrated under reduced pressure and precipitated in cold diethyl ether. The crude product was then washed with saturated sodium bicarbonate and followed by liquid–liquid extraction with DCM. The liquid–liquid extraction was repeated for 3 cycles. At each extraction cycle, the organic layer was collected and dried over anhydrous magnesium sulfate. Then, the solution was filtered and concentrated under reduced pressure. The purified product was then dried in a thermostated oven at 40 °C for 24 h. The purified product was obtained as a white powder in 92% yield with 90% conversion efficiency. ^1^H-NMR (700 MHz, CDCl_3_, 25 °C): δ 4.25 (s, 2H, *J* = 4.76 Hz, -CH_2_CO_2_-), δ 3.44 to 3.70 (br, -OCH_2_CH_2_O-), δ 3.31 (s, 3H, -OCH_3_), δ 1.87 (s, 6H, -CBr (CH_3_)_2_). GPC *M*_n_ = 7,402 g/mol; *M*_w_/*M*_N_ = 1.02.

#### Protection of 4-vinylphenylboronic acid

4-Vinylphenylboronic acid pinacol ester monomer was prepared according to the literature [19,20]. 4-Vinylphenylboronic acid (5.0 g, 33.8 mmol) and pinacol (4.0 mg, 34.0 mmol) were dissolved in anhydrous THF (100 ml) and stirred at room temperature for 2 h in air. The mixture was filtered and concentrated using a rotary evaporator, yielding a light-yellow viscous liquid with an 86% yield. ^1^H-NMR (700 MHz, CDCl_3_, 25 °C): δ 7.67 (d, 2H, *J* = 7.63 Hz, Ar-H), δ 7.29 (d, 2H, *J* = 7.77 Hz, Ar-H), δ 6.60 (dd, 1H, *J_1_* = 10.90 Hz, *J_2_* = 17.50 Hz, -Ar-CH=CH_2_), δ 5.69 (d, 1H, *J* = 17.64 Hz, -CH=CH_2_-E), δ 5.16 (d, 1H, *J* = 10.90 Hz, -CH=CH_2_-Z), δ 1.22 (s, 12H, Ar-BO_2_(C(CH_3_)_2_)_2_ (pinacol)).

#### Synthesis of poly(ethyleneglycol)-*b*-[(poly(3-vinylbenzaldehyde)-*r*-poly (4-vinylphenyl boronate ester)) mPEG-*b*-(PVB-*r*-PVBE) amphiphilic block copolymer

The synthesis of the random copolymers was carried out using atom transfer radical polymerization (ATRP) [19–23]. In a typical ATRP procedure, the mPEG-Br macroinitiator (2.0 g, 0.4 mmol) was added into a pre-dried Schlenk flask equipped with a magnetic stirring bar and heated at 60 °C to melt the mPEG-Br. Subsequently, a 1:10 molar ratio of 3-vinylbenzaldehyde (254 μl, 2 mmol) and 4-vinylphenylboronic acid pinacol ester (4.6 g, 20 mmol) was added to the mPEG-Br macroinitiator with a final molar ratio of [I]:[3-vinylbenzaldehyde]:[4-vinylphenylboronic acid pinacol ester] = 1:5:50. The resulting mixture was stirred under nitrogen for 30 min. In a separate Schlenk flask, CuBr (58.0 mg, 0.4 mmol) was degassed via 3 cycles of evacuation and purging with nitrogen. PMDETA (84.0 μl, 0.4 mmol) dissolved in toluene (5.0 ml) was then added into the CuBr flask. The Cu/PMDETA catalyst was subsequently injected into the flask using a syringe into the Schlenk flask containing the mPEG-Br macroinitiator to initiate the ATRP. The reaction was conducted at 90 °C in an oil bath for 24 h. After completion, the reaction was quenched with cold water and diluted with THF at room temperature. The diluted solution was filtered through a column packed with neutral aluminum oxide to remove the catalyst and the resulting solution was concentrated under reduced pressure. The viscous liquid was precipitated into 300 ml of cold hexane. The precipitate was dried in an oven at 40 °C for 24 h to yield a white powder with 83% yield. Eighty percent monomer conversion of 4-vinylphenylboronic acid pinacol ester and 3-vinylbenzaldehyde was calculated via ^1^H-NMR before being precipitated into cold hexane. ^1^H-NMR (700 MHz, CDCl_3_, 25 °C): δ 9.50 to 9.82 (br, CHO), δ 7.27 to 7.70 (br, Ar-H), δ 6.08 to 6.85 (br, Ar-H), δ 3.52 to 3.76 (br, -OCH_2_CH_2_O-), δ 3.38 (s, 3H, -OCH_3_), δ 1.59 to 1.67 (br, -CH_2_-CH-Ar), δ 1.14 to 1.40 (br, Ar-BO_2_(C(CH_3_)_2_)_2_ (pinacol). GPC *M*_n_ = 14,466 g/mol; *M*_w_/*M*_N_ = 1.04.

#### Self-assembly of polymer micelles

The preparation of the micelles involved dissolving 120 mg of mPEG-b-(PVB-r-PVBE) block copolymer (molecular weight: 14,466 g/mol) in 2 ml of THF. This solution was stirred until clear and then added dropwise to 4 ml of water while undergoing probe sonication [24]. The mixture was transferred to a 20-ml vial and left in a fume hood overnight for THF evaporation. Post-evaporation, the sample was vacuumed for 20 min to completely remove the THF. The final concentration of polymer was measured to be 30 mg/ml in water. A 2-ml aliquot of the aqueous polymer solution was then diluted with 10 ml of water to achieve a 5 mg/l concentration. Finally, the solution was frozen at −20 °C and lyophilized, yielding a 70% yield of white powder.

#### In vitro cytotoxicity assay

B16-F10 melanoma cells were seeded in a 96-well plate at a density of 7.0 × 10^3^ cells/well and allowed to incubate for 24 h to promote cell attachment and growth. MTS assay was conducted to access the cytotoxicity of the polymeric micelles and BPA toward the B16-F10 melanoma cells, following the protocol from the manufacturer. The micelle solution was diluted with the culture medium to obtain various concentrations of 1,000, 500, 250, 125, 62.5, 31.3, 15.6, and 0 μg/ml. Subsequently, the B16-F10 cells were then treated with the micelle solution and BPA at the respective concentrations for a duration of 24 h. Following this incubation, 20 μl of the MTS assay reagent was added to each well. The plate was then incubated for an additional 2 h at specific time intervals of 24, 48, and 72 h. Finally, the absorbance at 490 nm was measured using a plate reader to quantify the cell viability.

#### In vitro boron uptake

In vitro cellular uptake of boron-contained nanoparticles was conducted following our established protocol [25–27]. To visualize the cellular uptake of free anti-PD-L1 [28], B16-F10 melanoma cells were seeded on a round glass coverslip in a 24-well plate at a density of 2.0 × 10^4^ cells/ml and cultured overnight. The cells were then treated with free anti-PD-L1 (200 μg/ml) for 6 h and 12 h. After rinsing with phosphate buffered saline (PBS), the cells were fixed with 4% paraformaldehyde (formalin) and permeabilized with Triton X-100 for 10 min [29]. Subsequently, the cells were incubated with 20 μg/ml of FITC-conjugated goat anti-rat IgG (H+L) secondary antibody, 1 μg/ml of FAST-Dil, and 1 μg/ml DAPI. Following incubation, the staining reagent was thoroughly washed with PBS buffer. The cells were treated with an optical clearing agent that has been utilized to enhance the imaging depth and contrast of cell visualization and were covered with glass coverslips for fluorescence imaging.

To determine the intracellular uptake of boron by the B16-F10 melanoma cells, ICP-MS analysis was performed. Briefly, the B16-F10 melanoma cells (at a concentration of 3.0 × 10^5^/ml) were cultured in a 6-well plate overnight. The cells were incubated with 1 mg/ml BPA and 1 mg/ml mPEG-*b*-(PVB-*r*-PVBE) micelle for 12 h. Subsequently, the cells were rinsed with PBS to remove free boron drugs, followed by trypsinization. The cells were then dissolved using concentrated nitric acid and hydrofluoric acid, and the intracellular boron content was measured by ICP-MS.

#### In vitro BNCT treatment

B16-F10 melanoma cells were plated in a 96-well plate at a density of 7.0 × 10^3^ cells/well and cultured for 24 h [30]. Subsequently, the cells were then incubated with 1 mg/ml BPA and mPEG-*b*-(PVB-*r*-PVBE) micelles, respectively, for 12 h prior to irradiation. After the incubation period, the cells were rinsed twice with PBS, and the 96-well plates were placed onto a custom-made acrylic holder covered with a PE board. The PE board was utilized to decelerate the neutron flux to achieve a higher proportion of epithermal neutrons (0.5 eV to 10 keV) for neutron irradiation [26]. The neutron irradiation was conducted at a flux of 1 × 10^9^ neutrons/cm^2^⋅s and an irradiation power of 1.2 MW for 30 min, following our previous studies [25,26]. Following neutron irradiation, the cells were further incubated for 24 h and 48 h. Subsequently, 10 μl of MTS assay was added to each well and the plate was incubated for an additional 2 h. The absorbance was measured at a wavelength of 490 nm using a spectrometer to determine the cell viability after BNCT treatment.

#### In vivo tumor models

Male C57BL/6JNarl mice at 4 weeks of age were utilized to establish the B16-F10 melanoma mouse model. B16-F10 melanoma cells were cultured in DMEM supplemented with 10% FBS at 37 °C in a 5% CO_2_ incubator. The cells were harvested during the exponential growth phase, and the cell count was determined through a trypan blue exclusion assay. A total of 1 × 10^6^ cells in 50 μl of PBS were subcutaneously injected into the right hindlimb of the mice using a 26-gauge syringe. The tumor size was measured with a vernier caliper and tumor volume was calculated using Eq. 1:

$$\text{Volume}\;\left({\text{mm}}^{3}\right)=\frac{\left(a\times b^{2}\right)}{2}$$ (1)

where *a* and *b* are the length and width of the tumor, respectively. Animals were euthanized when the tumor size exceeded 1,000 mm^3^.

#### In vivo biodistribution of boron content

The in vivo experimental procedures were carried out by following the guidelines of the Institutional Animal Care and Use Committee (IACUC) at the Laboratory Animals Center at National Tsing Hua University (approval number: IACUC: 112002-1). The tumor implantation and grouping procedures were conducted following the literature [31]. After 7 days of tumor post-inoculation, the tumor volume of the mice reached approximately 100 mm^3^_._ The tumor-bearing mice were randomly divided into 2 groups, with each group consisting of 3 mice (*n* = 3). The mice were administered BPA and mPEG-*b*-(PVB-*r*-PVBE) micelle via intravenous injection at a concentration of 100 mg/kg every 3 days for a total of 3 doses. Following 24 h after the last injection, the mice were euthanized, and their organs and tumors were collected. Blood samples were collected using a 30-gauge syringe. All collected tissue samples, including the heart, liver, spleen, lung, kidney, and tumor, were weighed immediately. Subsequently, the samples were digested with a mixture of 68% nitric acid and hydrofluoric acid and heated at 60 °C for 2 h. The boron concentrations in the collected tissue samples from mice were determined using inductively coupled plasma mass spectrometry (ICP-MS).

#### In vivo BNCT and immunotherapy treatment

The tumor implantation and grouping procedures were conducted following the literature [31]. After 7 days of tumor post-inoculation, the tumor volume reached approximately 100 mm^3^. Subsequently, the mice were randomly divided into 4 groups (*n* = 3): non-irradiated control, neutron only, mPEG-*b*-(PVB-*r*-PVBE) micelle + neutron, and mPEG-*b*-(PVB-*r*-PVBE) micelle + neutron + immunotherapy with free anti-PD-L1. A total of 3 doses of mPEG-*b*-(PVB-*r*-PVBE) micelle were administered intravenously at a concentration of 100 mg/kg every 3 days. After 24 h following the final injection, the mice were placed in a customized acrylic holder (Fig. S1). The body and tail of the mice were secured with paper tape, and the tumor on the right hindlimb was immobilized toward the center of the holder. Subsequently, a PE board designed to decelerate the neutron flux to achieve a higher proportion of epithermal neutrons (0.5 eV to 10 keV) for neutron irradiation was used to cover the holder (Fig. S1) [32]. Before neutron irradiation, the mice were anesthetized intraperitoneally with 0.04 ml of Zoletil/Rompun cocktail (mixing ratio with Zoletil [50 mg/ml]/Rompun [23.32 mg/ml] = 1:4). The mice were irradiated with neutron irradiation at a flux of 1 × 10^9^ neutrons/cm^2^⋅s and an irradiation power of 1.2 MW for 30 min following our previous studies [25,26]. The mice were removed from the irradiation chamber and placed into a cage to allow the mice to recover from anesthesia. After 24 h of the neutron irradiation, the mice in the combination therapy group received intravenous injections of free anti-PD-L1 at a concentration of 20 mg/kg every 3 days for a total of 3 doses, respectively. Tumor sizes were measured 24 h before the BNCT treatment, designated as day zero.

#### Immunohistochemistry staining

For both hematoxylin and eosin (H&E) staining and immunohistochemical analyses, organ tissues (comprising liver, lung, and spleen), as well as tumor specimens, were initially fixed in 4% paraformaldehyde for a duration of 24 h, followed by a sequential dehydration process in graded ethanol solutions. Sections measuring 5 μm in thickness were obtained from the resulting paraffin-embedded samples. Subsequent to fixation, these sections were deparaffinized and rehydrated according to a standardized protocol. Staining was conducted either with H&E (Product Code: ab245880, Abcam) or with specific antibodies targeting CD3 (Product Code: 14-0032-81, eBioscience, Invitrogen), CD4 (Product Code: 14-0042-82, eBioscience, Invitrogen), and CD8a (Product Code: 14-0081-82, eBioscience, Invitrogen). Visualization was achieved through horseradish peroxidase/3,3′-diaminobenzidine (HRP/DAB) detection, utilizing the HRP/DAB (ABC) Detection IHC Kit (Product Code: ab64261, Abcam).

#### Statistical analysis

Data analyses were performed using 2-way analysis of variance (ANOVA) test to evaluate statistical significance between different experimental groups. Statistical analyses were performed using statistical software (SPSS 12.0, SPSS Inc. Chicago, IL). The figures were denoted with the statistical significance of *P < 0.05; **P < 0.01; ***P < 0.001.

## Results and Discussion

The amphiphilic block copolymers poly(ethylene glycol)-*b*-[(poly(3-vinylbenzaldehyde)-*r*-poly(4-vinylphenylboronate ester)] (mPEG-*b*-(PVB-*r*-PVBE)) were synthesized via a controlled radical polymerization technique. This method enabled precise control over the composition of the poly(4-vinylphenylboronate ester) (PVBE) repeating units [16]. Moreover, the benzaldehyde moieties were installed for potential conjugation with targeting ligands, antibodies, drugs, and imaging contrast agent. The synthesis of the amphiphilic mPEG-*b*-(PVB-*r*-PVBE) block copolymer is illustrated in Fig. 1. The first step in the preparation of the amphiphilic block copolymer mPEG-*b*-(PVB-*r*-PVBE) involved the synthesis of mPEG-Br macroinitiator starting from the methoxy end-functionalized PEG (MeO-PEG-OH) [19]. The synthesis of polymers containing boronic acid can be challenging due to factors such as hygroscopicity, instability [33], and the propensity for undesired formation of boroxine species during reactions [34]. A major obstacle in this synthesis is the acidic nature of the boronic acid moiety, which can hinder polymerization reactions, particularly those dependent on metal complexes as catalysts, such as ATRP. The boronic acid can oxidize or form complexes with the metal catalysts used in ATRP, resulting in diminished control over the polymerization process [35]. To circumvent these challenges, a common approach is to shield the boronic acid groups prior to the polymerization. The synthesis of the mPEG-b-(PVB-r-PVBE) amphiphilic block copolymer was achieved by chain extending a mixture of 4-vinylphenylboronate ester (protected 4-vinylphenyl boronic acid) and 3-vinylbenzaldehyde from the mPEG-Br macroinitiator via ATPR. This process yielded a well-defined amphiphilic block copolymer mPEG-*b*-(PVB-*r*-PVBE) with *M*_n_ = 14,466 g/mol and a narrow polydispersity index (PDI) of 1.04 (Fig. 1). Subsequently, boron-rich polymer micelles were prepared through the self-assembly of this amphiphilic mPEG-*b*-(PVB-*r*-PVBE) block copolymers in a THF/water mixture, following the protocol reported in the literature [36].

### Characterization of mPEG-*b*-(PVB-*r*-PVBE) amphiphilic block copolymer

**Fig. 2.**
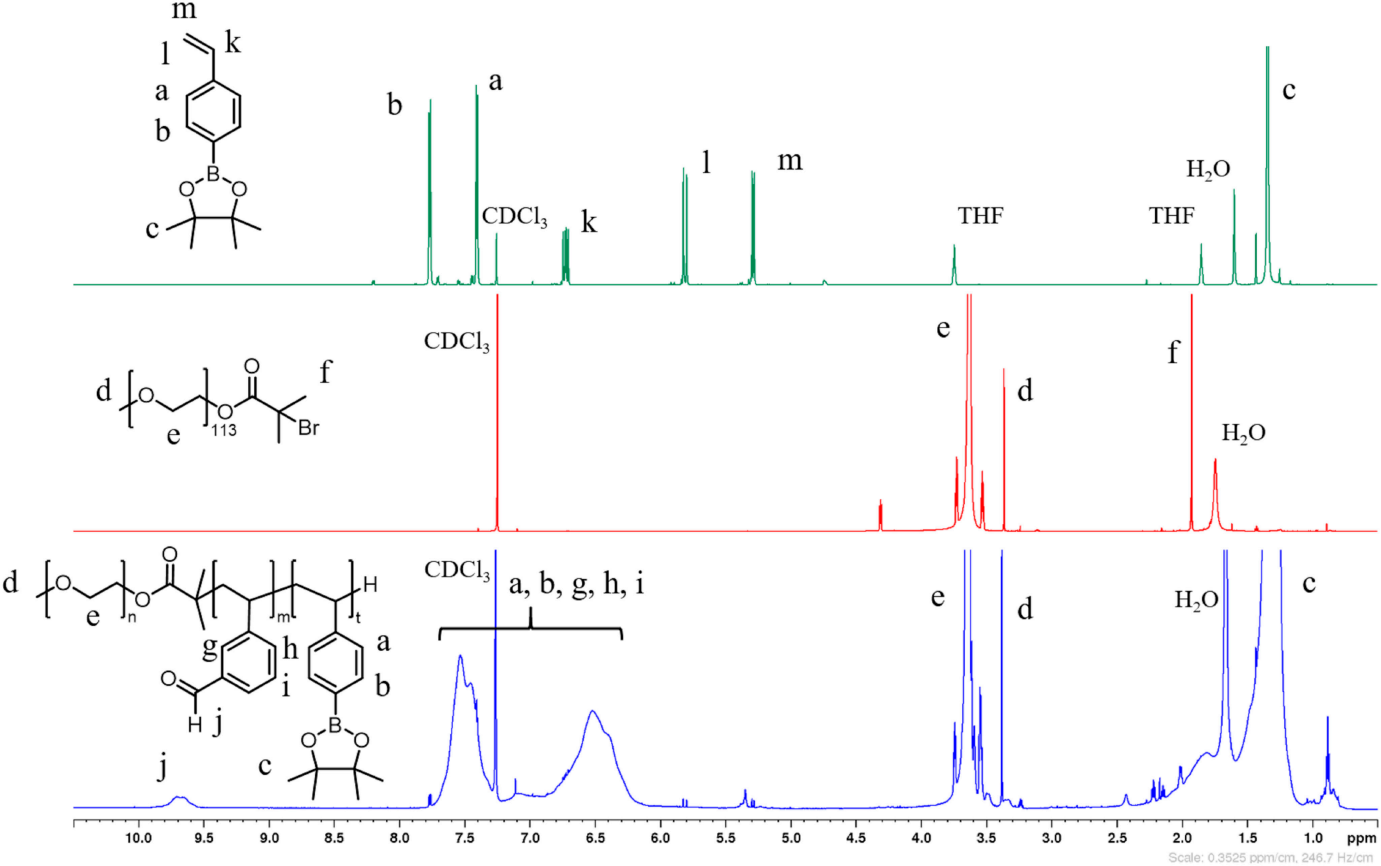
^1^H-NMR spectra of 4-vinylphenyl boronic acid pinacol ester, mPEG-Br macrorinitiator, and mPEG-*b*-(PVB-*r*-PVBE) amphiphilic block copolymer.

Figure 2 shows the stacked ^1^H-NMR spectrum of the 4-vinylphenylboronic acid pinacol ester monomer, mPEG-Br macroinitiator, and the mPEG-*b*-(PVB-*r*-PVBE) amphiphilic block copolymer. The successful protection of 4-vinylphenylboronic acid by pinacol groups, forming the 4-vinylphenylboronic acid pinacol ester, is indicated by a sharp resonance at 1.22 ppm. This peak corresponds to the protons of the pinacol methyl group (Ar-BO_2_(C(CH_3_)_2_)_2_). Additionally, the broad peak observed between 3.44 and 3.70 ppm can be assigned to the methylene protons of the repeating units of mPEG (-CH_2_-CH_2_-O). The sharp resonance at 3.31 ppm is attributed to the methoxy protons of mPEG (-O-CH_3_). Lastly, the 6 methyl protons adjacent to the bromine appeared at 1.87 ppm (-CBr(CH_3_)_2_). The esterification efficiency of α-bromoisobutyryl bromide onto mPEG-OH was calculated using the integration ratio of the methylene protons peaks of mPEG (-CH_2_-CH_2_-O) and the methyl protons peaks adjacent to the bromine.

$$\begin{array}{c} \text{Esterification efficiency}\;(\;\%\;)=\frac{\;I_{e}/113\times 4}{\;I_{f}/6}\times 100 \end{array}$$ (2)

Employing Eq. 2, where *I_e_* and *I_f_* are the integral values of the broad peaks at 3.44 to 3.70 ppm (H_e_, -CH_2_-CH_2_-O) and 1.87 ppm (H_f_, -CBr(CH_3_)_2_), respectively, the esterification efficiency was determined to be 90%. Starting from the mPEG-Br macroinitiator, the ^1^H-NMR spectrum of the resulting mPEG-b-(PVB-r-PVBE) block copolymer reveals new peaks, signifying a successful chain extension of PVB and PVBE from the mPEG macroinitiator. A small broad peak at 9.50 to 9.82 ppm aligns with the vinyl benzaldehyde protons (-CHO), while a broad peak at 1.14 to 1.40 ppm corresponds to the pinacol ester methyl protons (-BO_2_(C(CH_3_)_2_)_2_) emerged after the chain extension. These findings confirm that the successful incorporation of the 3-vinylbenzaldehyde moieties and the 4-vinylphenyl boronic acid pinacol ester segments onto the mPEG-Br is consistent with existing literature [37]. Finally, the degree of polymerization (DP) for both the PVB and PVBE segments was calculated using end-group analysis from the ^1^H-NMR spectrum. Utilizing Eqs. 3 and 4, where *I_e_*, *I_c_*, and *I_j_* are the integral values of the broad peak between 3.44 and 3.70 ppm (H_e_, -CH_2_-CH_2_-O), the broad peak at 1.14 to 1.40 ppm (H_c_, -BO_2_(C(CH_3_)_2_)_2_), and the small peak at 9.50 to 9.82 ppm (H_j_, -CHO), respectively, the DP was calculated to be 4 for the polyvinylbenzaldehyde and 40 for the polyvinylphenylboronic acid pinacol ester (PVBE), respectively.

$$\text{Degree of polymerization}\;\left(\text{DP}\right)\;\text{of the PVBE}=\frac{\;I_{j}/1\;}{\;I_{e}/113\times 4}\;$$ (3)

$$\text{Degree of polymerization}\;\left(\text{DP}\right)\;\text{of the}\;\text{PVB}=\frac{\;I_{c}/12\;}{\;I_{e}/113\times 4}$$ (4)

### GPC of amphiphilic mPEG-*b*-(PVB-*r*-PVBE) block copolymer

The chain extension of amphiphilic poly(PVB-*r*-PVBE) copolymer from mPEG-Br was confirmed by gel permeation chromatography conducted in THF, as shown in Fig. S2. The gel permeation chromatogram profile of the mPEG-*b*-(PVB-*r*-PVBE) clearly demonstrates an increase in *M*_n_ from 7,402 g/mol for the mPEG-Br macroinitiator to 14,466 g/mol for the synthesized block copolymer. Notably, the increase in molecular weight was achieved while maintaining a narrow low PDI of 1.04, which suggests that the chain extension from the mPEG-Br macroinitiator to the mPEG-b-(PVB-r-PVBE) block copolymer was well-controlled. Such control is essential for ensuring the uniformity and consistency of the copolymer’s properties, which are critical factors in its potential applications, particularly in the field of targeted drug delivery and cancer therapy.

### Size distribution and TEM morphology

**Fig. 3.**
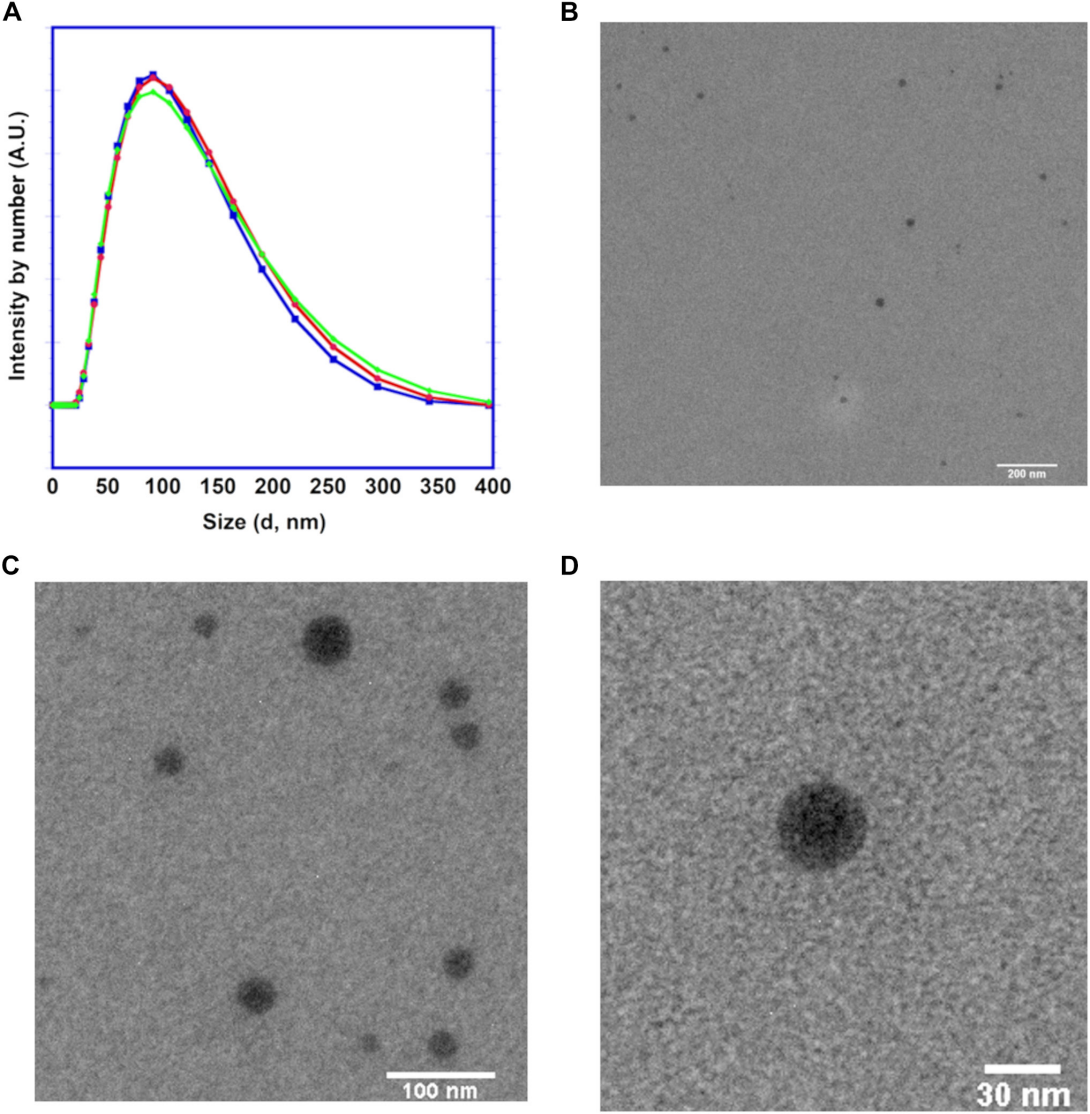
(A) DLS hydrodynamic size, and (B to D) TEM images at different magnifications of the mPEG-*b*-(PVB-*r*-PVBE) micelles.

The average hydrodynamic diameter of the mPEG-*b*-(PVB-*r*-PVBE) micelles was determined by dynamic light scattering (DLS) (Fig. 3A), and their morphology was examined by transmission electron microscopy (TEM), as shown in Fig. 3B to D. The DLS analysis revealed that the average hydrodynamic size of the mPEG-b-(PVB-r-PVBE) micelles was 91.3 nm with a PDI of 0.18, as per number-weighted distribution. On the other hand, the TEM images showed that mPEG-*b*-(PVB-*r*-PVBE) micelles adopt a spherical shape with an average size of 26.5 ± 8.3 nm (*n* = 35). The differences in particle size measurement between DLS and TEM could be attributed to the inherent differences in principles underlying these techniques [38]. DLS measures the hydrodynamic size, which encompasses not only the micelle core but also the solvated polymer corona along and surrounding solvent molecules. This often results in an apparent overestimation of the size. In contrast, TEM imaging occurs in an ultra-high vacuum environment, which may lead to the collapse of the soft polymer micelles, thus reflecting a smaller size [39]. Both these measurements together offer a holistic view of the micellar architecture, essential for the development of effective nanocarriers in cancer therapy.

### In vitro cytotoxicity of polymeric micelles

**Fig. 4.**
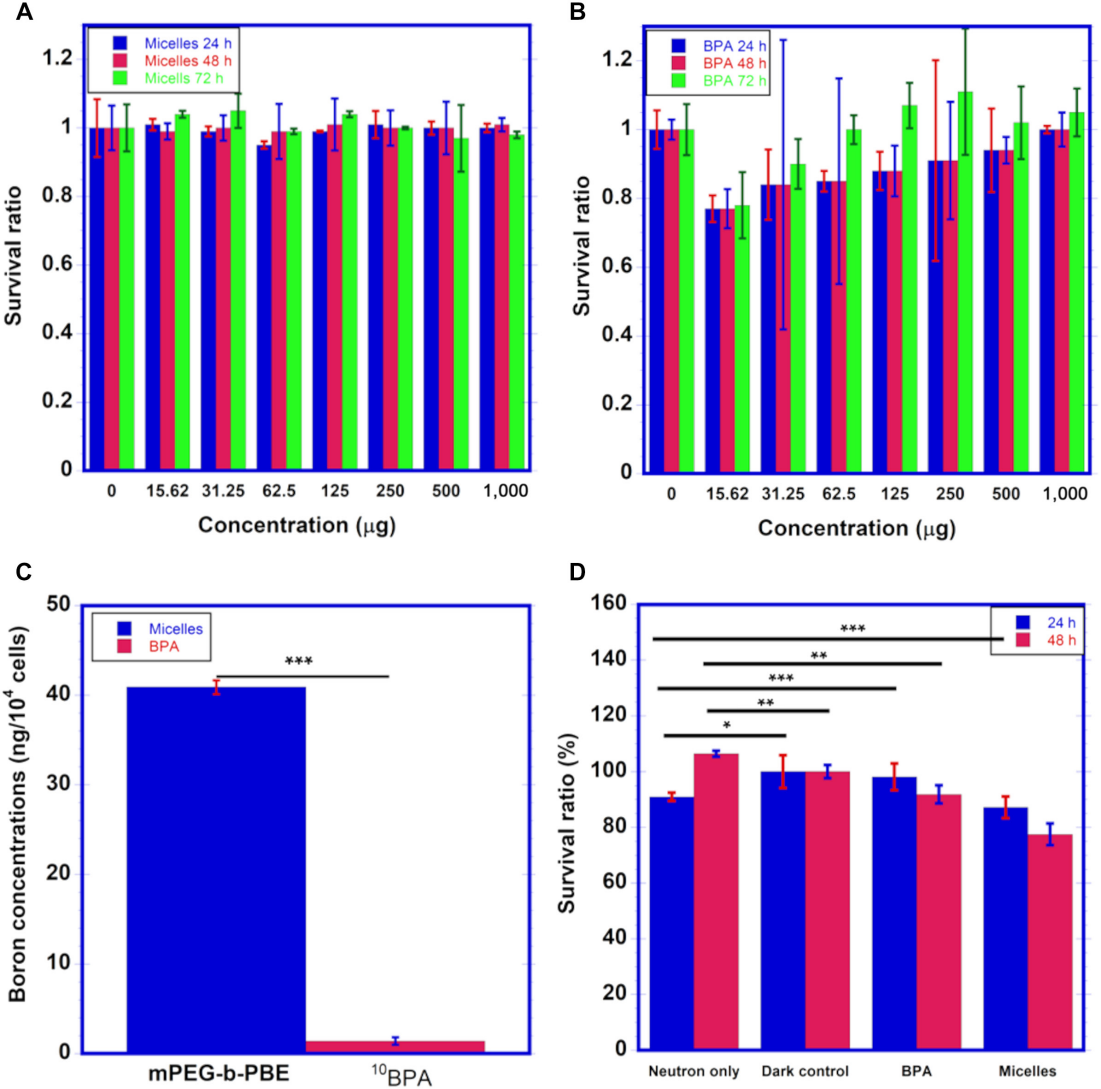
Survival ratio of B16-F10 melanoma cells determined by MTS assay at 24, 48, and 72 h upon treatment with boron drugs (A) mPEG-*b*-(PVB-*r*-PVBE) micelles and (B) BPA with concentration gradient from 0 to 1,000 μg/ml of solutions. (C) Boron concentration in B16-F10 melanoma cells treated with mPEG-*b*-(PVB-*r*-PVBE) micelle and BPA, determined by ICP-MS analysis. (D) Cell viability of B16-F10 melanoma cells after treatment with 1 mg/ml BPA and polymer micelles for 12 h, followed by a 30-min neutron irradiation. The cell viability was assessed using the MTS assay at 24-h and 48-h post-BNCT treatment. *P < 0.05, **P < 0.01, ***P < 0.001 (2-way ANOVA).

The cell viability of B16-F10 melanoma cells was evaluated using the MTS assay following treatment with mPEG-*b*-(PVB-*r*-PVBE) micelle and BPA (Fig. 4). The B16-F10 melanoma cells were treated to varying concentrations of mPEG-*b*-(PVB-*r*-PVBE) micelles and ranging from 0 to 1,000 μg/ml, over periods of 24, 48, and 72 h. As illustrated in Fig. 4A, at lower micelle concentrations (15.6, 31.3, and 62.5 μg/ml), cell viability remained nearly 100%, suggesting negligible cytotoxicity at these concentrations. At higher concentrations of the micelles (125, 250, 500, and 1,000 μg/ml), an increasing trend in cell viability was observed from 24 to 72 h. This trend implies that initial drug-induced cell death was followed by subsequent cell proliferation over time, leading to cell viability levels of 100% or even higher. This phenomenon indicates a potential compensatory proliferation response triggered by the initial cytotoxic effect. Similarly, Fig. 4B displays the cytotoxic effect of BPA on B16-F10 melanoma cells. At lower concentrations (15.6, 31.3, 62.5, 125, and 250 μg/ml), cell viability was maintained at approximately 90% after 48 h of incubation. Conversely, higher concentrations of BPA (500 and 1,000 μg/ml) resulted in markedly reduced viability within the first 24 h. Interestingly, like the polymer micelles, prolonged incubation with BPA led to cell viabilities exceeding 100%, due to increased cell proliferation, a phenomenon also reported in other studies [40]. From these results, it is evident that the mPEG-b-(PVB-r-PVBE) micelles are non-toxic to B16-F10 melanoma cells, maintaining 100% survival at a concentration of 1 mg/ml over a 72-h incubation period. Additionally, the mPEG-*b*-(PVB-*r*-PVBE) micelles exhibit substantially lower cytotoxicity compared to BPA at high concentrations. This suggests that higher drug concentrations, inherently containing more boron content, can be utilized during treatment.

### In vitro boron uptake of B16-F10 melanoma cells

The quantitative measurement of boron uptake in B16-F10 melanoma cells was conducted using ICP-MS, comparing the cellular accumulation of boron from mPEG-b-(PVB-r-PVBE) micelles and BPA. As depicted in Fig. 4C**,** the mPEG-*b*-(PVB-*r*-PVBE) micelles demonstrated a progressive increase in boron accumulation within the melanoma cells over extended incubation periods. The cellular uptake and retention of boronophenylalanine (BPA) present a stark contrast to that of mPEG-b-(PVB-r-PVBE) amphiphilic block copolymers. Existing literature indicates that BPA reaches its peak accumulation in cells at approximately 2 h post-administration [41], followed by active efflux as the concentration gradient of extracellular amino acids diminishes. This necessitates continuous administration of BPA during BNCT to maintain a high boron concentration in the bloodstream [8]. In contrast, mPEG-b-(PVB-r-PVBE) amphiphilic block copolymers, with an optimal size of approximately 85.5 nm and a hydrophilic polymer corona, exhibit a preferential accumulation in tumor sites. This is attributed to the EPR effect [42], a phenomenon where the increased permeability of tumor vasculature and the elevated interstitial fluid pressure (IFP) at the tumor sites facilitate the passive targeting and accumulation of nanodrugs. By exploiting the EPR effect, these copolymers could overcome the limitations associated with BPA, ensuring increased boron accumulation and prolonged retention of boron drugs within tumor cells.

### In vitro cell viability after BNCT treatment

The in vitro BNCT treatment was conducted to assess the efficacy of mPEG-*b*-(PVB-*r*-PVBE) micelle as a tumor-killing agent. B16-F10 melanoma cells were treated with BPA and mPEG-*b*-(PVB-*r*-PVBE) micelle at a concentration of 1 mg/ml for 12 h, followed by 30 min of neutron irradiation. Figure 4D illustrates the cell viability results, determined by the MTS assay, at 24 h and 48 h post-BNCT treatment. In the neutron-only control group, approximately 10% cell death was observed 24 h post-treatment. This cell apoptosis can be attributed to the low-energy LET γ-rays emitted from the neutron capture fission reaction during BNCT treatment [43]. These γ-rays induce the production of reactive oxygen species (ROS) [44], leading to oxidative damage to healthy tissues [45]. The group treated with BPA exhibited 100% cell viability 24 h post-BNCT. However, a notable 10% reduction in cell viability was observed at 48 h post-BNCT. This pattern reflects the limited efficacy of BPA in BNCT, primarily due to its short retention time and the limited B-10 accumulation within the cells. As reported in the literature [41], BPA is rapidly effluxed from the cells by the antiport mechanism as the concentration gradient of the extracellular amino acids decreases. This rapid efflux results in a reduced accumulation of BPA in cells, thereby reducing its effectiveness as a BNCT agent. In contrast, cells treated with mPEG-*b*-(PVB-*r*-PVBE) micelle displayed a 2-fold reduction in cell viability 48 h post-BNCT when compared to the BPA-treated group. This significant decrease in viability indicates the superior efficacy of the mPEG-b-(PVB-r-PVBE) micelles in delivering B-10 into cancer cells and thus enhanced the overall efficacy of BNCT. Compared to the BPA-treated group, the mPEG-*b*-(PVB-*r*-PVBE) micelle group demonstrated more effective accumulation and retention within the cells, as corroborated by ICP-MS analysis of boron uptake. This is attributed to the EPR effect, which allows for the higher accumulation and retention of nanodrugs at the tumor site [46]. Therefore, following BNCT treatment, a marked reduction in tumor cell viability was observed in the mPEG-b-(PVB-r-PVBE)-treated group, indicating superior efficacy compared to BPA. This outcome aligns with literature findings that the effectiveness of BNCT relies on the accumulation of B-10 within the tumor cells, rather than the characteristic of the neutron beam [7]. These results highlight the potential of mPEG-b-(PVB-r-PVBE) micelles as an effective agent for BNCT, offering improved therapeutic outcomes due to their enhanced cellular uptake and retention capabilities.

### In vivo biodistribution of boron content in melanoma tumor-bearing mice

**Fig. 5.**
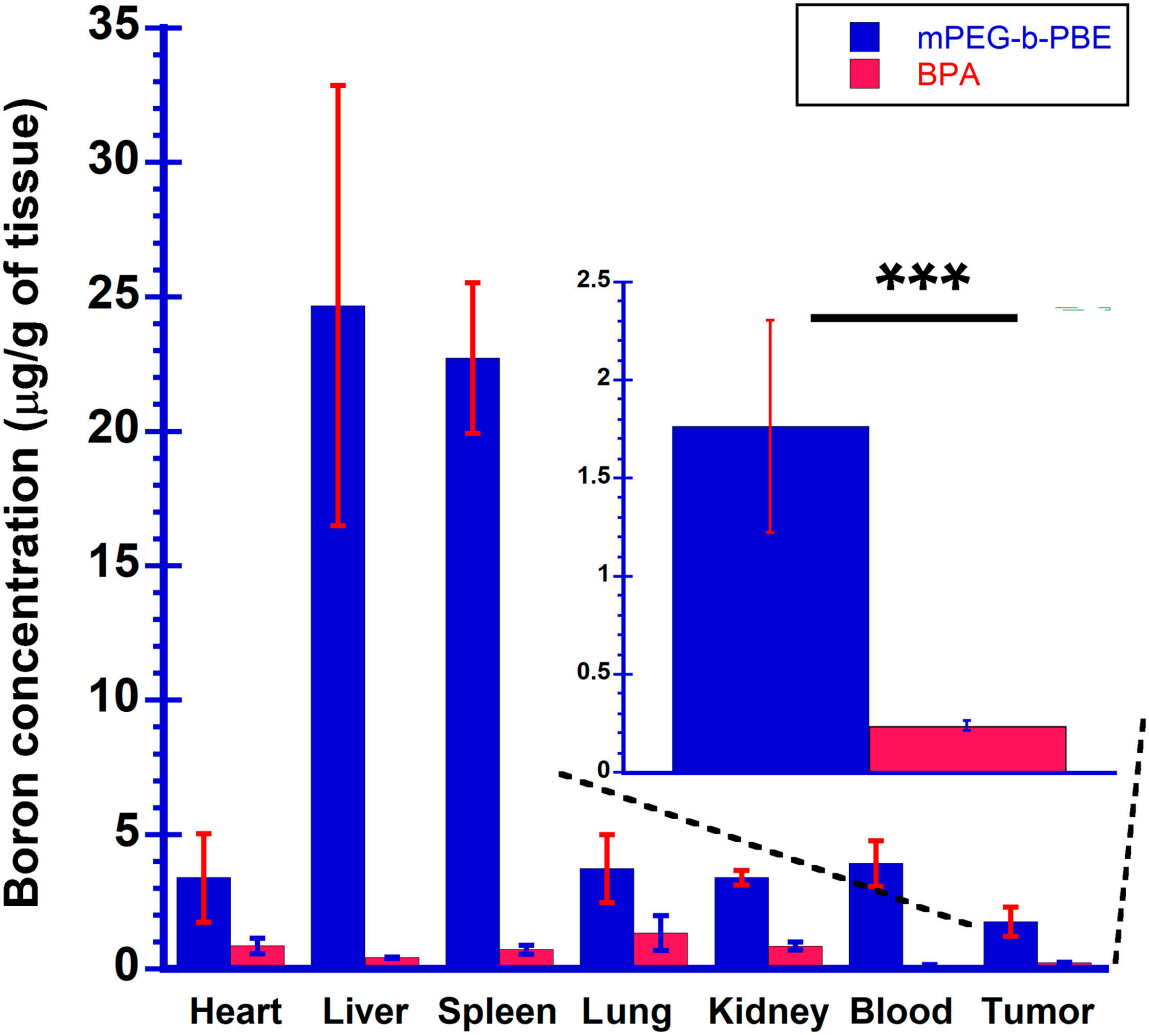
In vivo biodistribution of boron content in B16-F10 melanoma tumor-bearing mice (*n* = 3). The mice were intravenously injected with BPA and mPEG-*b*-(PVB-*r*-PVBE) micelle at a concentration of 100 mg/kg every 3 days for a total of 3 doses. The boron contents in the tumors and organs were quantified using inductively coupled plasma mass spectrometry (ICP-MS). ***P < .0.001 (2-way ANOVA).

In vivo biodistribution of the BPA and mPEG-*b*-(PVB-*r*-PVBE) micelles was conducted in B16-F10 melanoma tumor-bearing mice to study the distribution of the boron drug in the mice. The boron content in various organs was measured using ICP-MS in various organs, tumors, and blood samples. Figure 5 shows that the highest boron accumulation was in the liver and spleen, which is consistent with other nanoparticle-based drug delivery agents [47]. The boron accumulation in the tumors was observed to be higher in mice treated with the mPEG-*b*-(PVB-*r*-PVBE) micelles than in the harvested tumor in mice treated with BPA (***P < 0.001). This result may be attributed to the rapid clearance of BPA within 6 h as described in the literature [41]. Our study here suggests that these boron-rich micelles can effectively deliver boron-10 to the tumor tissue, resulting in the enhanced accumulation of boron in the tumor tissue for improved therapeutic efficacy of BNCT treatment.

### In vivo synergistic therapy of BNCT and immunotherapy

**Fig. 6.**
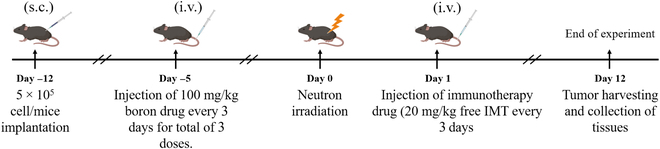
Schematic strategy of tumor model construction and combinatorial treatment strategies.

**Fig. 7.**
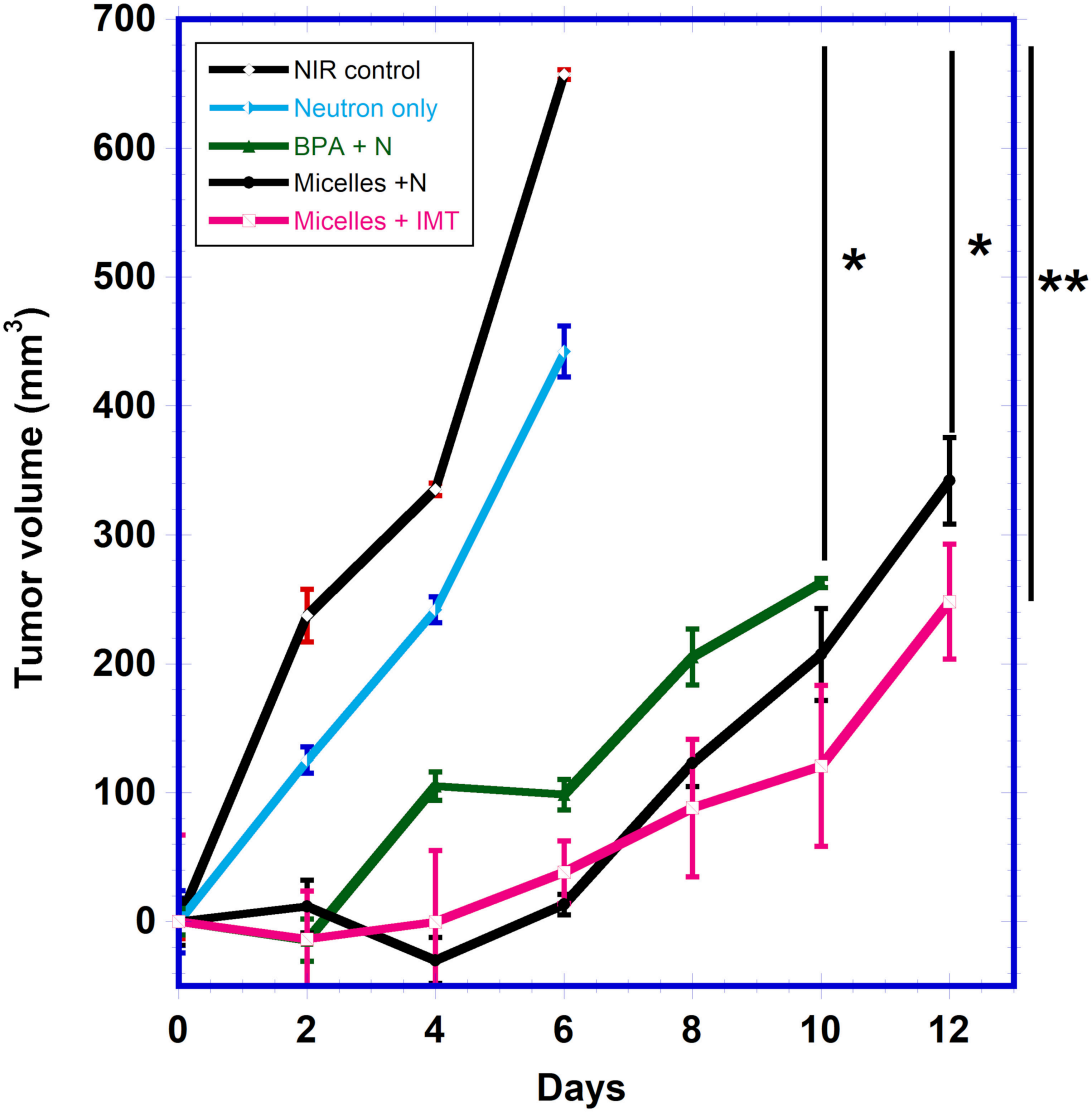
Tumor growth curve (%) of tumor model according to the tumor volume of day 0. Comparison of tumor growth in mice treated with BPA (green curve) and mPEG-*b*-(PVB-*r*-PVBE) micelle (black curve) via BNCT monotherapy. The tumor growth delay in mice treated with mPEG-*b*-(PVB-*r*-PVBE) micelle + free anti-PD-L1 combined treatment is represented with a pink curve. Statistical significance is indicated as follows: *P < 0.05 and **P < 0.01 as determined by 2-way ANOVA.

In this in vivo study, the tumor suppression efficacy of 2 treatment modalities was accessed: BNCT monotherapy and in combination with immunotherapy (Figs. 6 and 7). The results revealed that the combined therapy demonstrated greater inhibition of tumor growth compared to mice in the control group. The TGD was utilized as a metric, calculated based on the difference in doubling time (DT) between untreated and treated tumors, defined as (TGD (days) = DT_tr_ − DT_untr_). First, DT of the non-irradiated control group was calculated according to Eq. 5:

$${\text{DT}}_{\text{untr}}\left(\text{days}\right)=\frac{\ln2}{\beta }$$ (5)

where DT_untr_ is the solution to the equation *V*_0_*e^βt^* = 2*V*_0_, *β* representing the slope of the logarithmic tumor growth curve for the non-irradiated control group. DT_untr_ was calculated to be = ln 2/0.33 = 2.1 days. For the treated groups, DT was calculated according to Eq. 6:

$${\text{DT}}_{\text{tr}}\left(\text{days}\right)=\frac{\alpha -\gamma +\ln2}{\beta }$$ (6)

where *α* was defined as ln*V*_0_ for each group, *β* represents the slope of the logarithmic tumor growth curve, and *γ* denotes the intercepts of these curves. The results are presented in Table.

**Table.** Comparative analysis of tumor growth delay across treatment groups. In this table, α represents the logarithm of tumor volume at day 0; γ signifies the intercepts of logarithm scale linear function describing tumor growth; β is indicative of the slope of this logarithmic growth function, DT_tr_ denotes the doubling time of the tumors post-treatment, and TGD is the difference in doubling times between of treated and untreated tumors.

Group	α	γ	β	DT (days)	TGD (days)
1. Non-irradiated control	-	-	0.33	2.1	-
2. Neutron only	5.66	5.77	0.27	2.16	0.1
3. BPA+N	5.73	5.61	0.15	5.42	3.3
4. Micelle+N	5.58	5.23	0.14	7.45	5.4
5. Micelle+N+free IMT	5.87	5.61	0.11	8.66	6.6

N, neutron irradiation; free IMT, free anti-PD-L1.

The efficacy of BNCT monotherapy in suppressing tumor growth was examined by comparing the TGD in mice treated with mPEG-*b*-(PVB-*r*-PVBE) micelle + N to those treated with BPA + N, neutron only, and the non-irradiated control group (Table). A significant reduction in tumor growth was observed in the mPEG-*b*-(PVB-*r*-PVBE) micelle + N treatment group compared to the non-irradiated control groups (*P < 0.05), as shown in Fig. 7 and Table. The mPEG-*b*-(PVB-*r*-PVBE) micelle exhibited a TGD of 5.4 days, surpassing the TGD of 3.3 days in the BPA-treated group. This enhancement is attributed to the higher boron accumulation of the mPEG-*b*-(PVB-*r*-PVBE) micelle (1.64 μg of boron/g of tissues) within the tumor, in comparison to BPA (0.57 μg of boron/g of tissues) according to our biodistribution results shown in Fig. 5. Furthermore, the study accessed the effectiveness of combined immune-neutron therapy using mPEG-*b*-(PVB-*r*-PVBE) micelle against BNCT monotherapy (Fig. 7, red curve). The TGD of the combined therapy group extended from 5.4 to 6.6 days in the combined therapy group (Table). This enhancement in TGD is attributed to the synergistic effect of the mPEG-b-(PVB-r-PVBE) micelles in conjunction with immune checkpoint blockade therapy. Initially, the boron-rich polymer micelles facilitate effective BNCT, directly targeting and destroying tumor cells. Subsequently, the BNCT is complemented by blocking the PD-L1 immune checkpoint on the surface of cancer cells’ surface, reactivating the host immune system. This synergistic approach not only addresses the selective destruction of tumor cells but also harnesses the body’s natural defense mechanisms to provide a more comprehensive and potentially longer-lasting cancer treatment strategy.

**Fig. 8.**
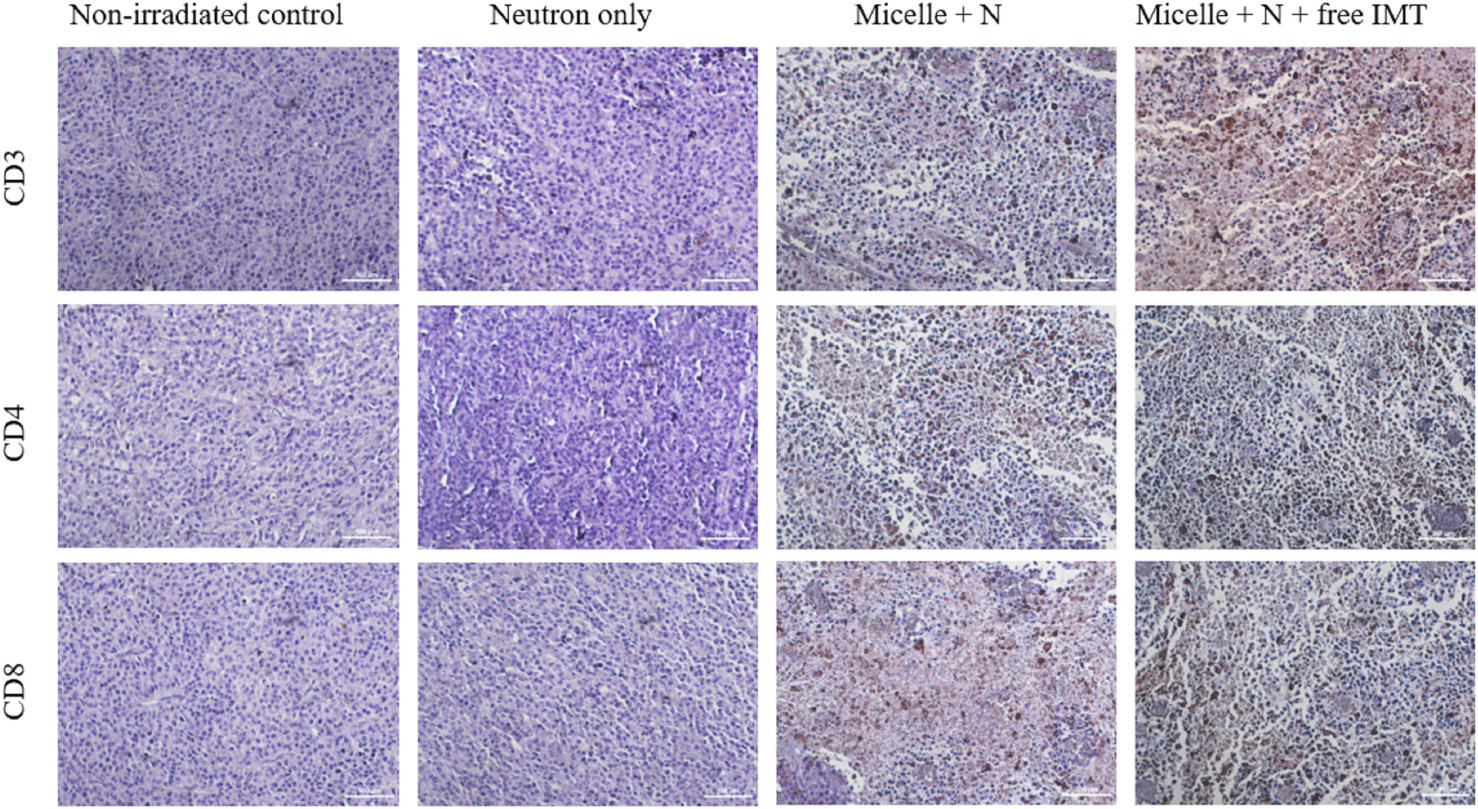
Immunohistochemical staining of B16-F10 tumor tissue sections using CD3, CD4, and CD8 antibodies to visualize T-cell subpopulations within the tumor microenvironment. The CD3 antibody is used to reveal the overall distribution of T cells, the CD4 antibody highlights the presence of helper T cells, and the CD8 antibody identifies cytotoxic T cells within the tumor microenvironment. Scale bar = 100 μm.

Subsequently, we evaluated the distribution of T-cell populations within tumor tissues by staining using CD3, CD4, and CD8 antibodies on tumor tissue sections from mice in the prescribed experimental group (Fig. 8) [48]. First, a distinct brown color was observed in the tissue sections from both mPEG-*b*-(PVB-*r*-PVBE) micelle + N and mPEG-*b*-(PVB-*r*-PVBE) micelle + N + I (BNCT + immunotherapy) treatment groups, in comparison to the control group. This brown coloration indicates the aggregation or activation of CD3, CD4, and CD8α-positive T cells. The presence of these color changes suggests an active immune response, with both treatment groups showing evidence of immune cell infiltration and engagement within the tumor tissue. This is indicative of a heightened immune response against the tumor, potentially enhancing the therapeutic efficacy. Notably, a deeper coloration was observed in the mPEG-*b*-(PVB-*r*-PVBE) micelle + N + I treatment group compared to the mPEG-*b*-(PVB-*r*-PVBE) micelle + N. This result suggests an intensified response, indicating that the combination of immune checkpoint blockade therapy following BNCT treatment further enhanced the activation and infiltrations of T cells specifically to the tumor site. These findings corroborate with the TGD analyses, which imply that combined BCNT and immune checkpoint blockade approach, as applied in the mPEG-b-(PVB-r-PVBE) micelle + N + I group, may offer a synergistic effect, enhancing a more sustainable cancer immunity. Our findings also align with existing literature on the synergistic effects of radiotherapy and immunotherapy against various cancer types [49]. The observed synergistic effects can be attributed to the capacity of BNCT to induce ICD by leveraging the high LET ionizing radiation to destroy primary tumors. ICD triggers cytotoxic immune responses against the primary and distant tumors, characterized by the infiltration of cytotoxic T cells into the tumor microenvironment. Cancer cells, however, have developed mechanisms to evade the cancer–immunity cycle. This evasion is facilitated through the expression of PD-1 and CTLA-4 ligands on the T-cell surface, and PD-L1 on the surface of cancer cells, which transmit inhibitory signals that down-regulate T-cell activation [10]. Specifically, B16-F10 melanoma tumors are known to overexpress PD-L1 on the transmembrane protein of the cancer cells, initiating evasion of autoimmunity when PD-1 (expressed on T cells) and its ligand PD-L1 (expressed on cancer cells) interact with PD-L1 on cancer cells, forming a PD-1/PD-L1 axis [10]. These interactions between PD-1 and PD-L1 adversely regulate the immune response by decreasing the cytokine-producing signals and simultaneously inducing apoptosis of the T lymphocyte [50]. In the context of our study, mice treated with anti-PD-L1 following BNCT demonstrated effective blockade of the PD-1/PD-L1 signaling pathway. By reversing the immunosuppressive environment, this strategy promoted T-cell infiltration and activation at the tumor sites after the initial BCNT treatment, as evidenced by the T-cell population at the tumor tissues in mice treated with combined therapy (Fig. 8). As a result, mice receiving the combination therapy of immunotherapy post-BNCT exhibited greater TGD compared to those treated with BNCT alone (Fig. 7).

**Fig. 9.**
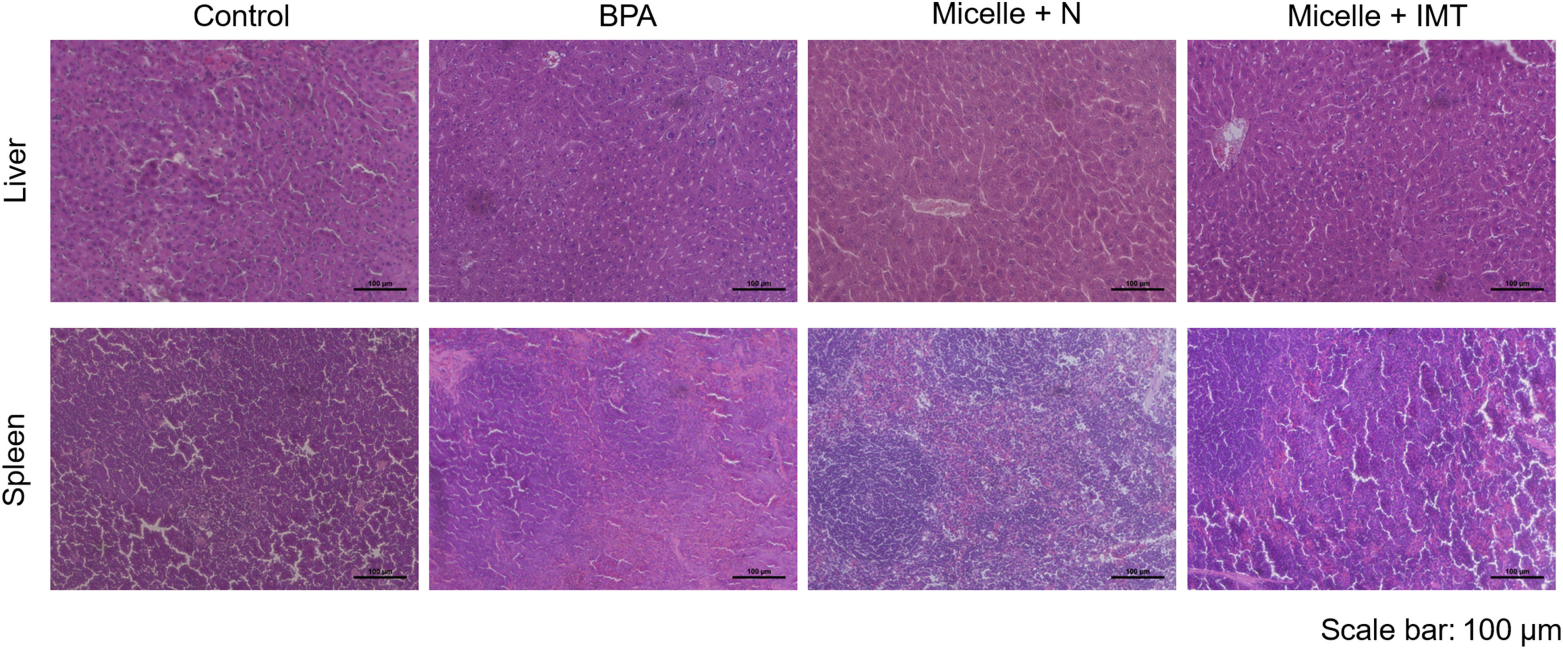
Immunohistochemical staining of the hematoxylin and eosin staining of the liver and spleen of mice in the control and experimental groups. Scale bar = 100 μm.

Lastly, we further demonstrated the specificity of BNCT in selectively killing cancer cells and evaluated the potential toxicity of the combined therapy. This assessment was conducted by monitoring the mice’s body weight throughout the treatment period. Firstly, we observed a rapid increase in body weight in both the control group due to the rapid tumor growth. Our findings also revealed that there is no significant weight loss in mice in the treatment group, which suggests that both the prescribed BNCT monotherapy and the combined therapy of BNCT and immune checkpoint blockade could offer highly effective and targeted treatment options for melanoma patients with minimal side effects (Fig. S3). To investigate the implications of polymer micelle accumulation in the liver and spleen during BNCT, we conducted a detailed histological examination of these organs in mice from various treatment groups, including the control, BPA-treated, micelle-treated with neutron irradiation (micelles + N), and micelle-treated with neutron irradiation in combination with immunotherapy (micelles + IMT). The analysis was carried out using H&E staining to assess any potential morphological changes or damage induced by the treatment. As presented in Fig. 9, the examination revealed no substantial morphological alterations or damage in the liver and spleen tissues of mice treated with polymer micelles, whether exposed to neutron irradiation alone or in combination with immunotherapy, when compared to the control group. These observations suggest that despite the pronounced accumulation of polymer micelles within these organs during BNCT, there were no detectable adverse effects on their structural or functional integrity. This finding underscores the biocompatibility and safety of utilizing polymer micelles as a drug delivery system in the context of BNCT, highlighting their potential for clinical application without adverse impact on organ health.

## Conclusion

In this study, we successfully synthesized boron-rich block copolymer micelles using amphiphilic PEG-b-PVBE block copolymers. These micelles, which possessed an optimal hydrodynamic diameter of 85.5 nm and a low PDI of 0.2, demonstrated a remarkable 13-fold increase in boron uptake in B16-F10 melanoma cells compared to the conventional boron drug BPA. This enhanced uptake, coupled with prolonged tumor retention, significantly improved the tumoricidal effectiveness of BNCT on melanoma cells. This was evidenced by the reduced cell viability post-irradiation (77%) in boron-rich polymer micelle-treated cells, as opposed to a higher viability (90%) in cells treated with BPA. Furthermore, in vivo studies conducted on melanoma tumor-bearing mice revealed that the boron-rich polymer micelles outperformed BPA in terms of tumor eradication and TGD. Specifically, the micelle-treated group experienced a TGD of 5.4 days, surpassing the 3.3-day delay observed with BPA treatment. More impressively, the integration of BNCT with anti-PD-L1 immunotherapy extended this TGD to 6.6 days, highlighting a synergistic effect. This combination therapy not only inhibited tumor growth more effectively than BNCT alone but also significantly boosted T-cell infiltration and activation at the tumor sites, suggesting an intensified immune response. In conclusion, our research findings demonstrate that the novel boron-rich polymer micelles substantially enhance BNCT efficacy. The synergistic combination of BNCT and immune checkpoint blockade results in improved tumor control and the potential for extended survival of cancer patients in the clinic. This study presents a promising therapeutic potential of integrating nanomedicine with cancer treatment strategies, opening new avenues for cancer treatment advancements.

## Data Availability

The data reported in the manuscript and Supplementary Material is available upon request from Pei Yuin Keng at keng.py@gapp.nthu.edu.tw.
