# Supplementary material for: Enhancing Cancer Therapy: Boron-Rich Polyboronate Ester Micelles for Synergistic Boron Neutron Capture Therapy and PD-1/PD-L1 Checkpoint Blockade
Source: Biomater Res. 2024 Jun 26;28:0040. doi: 10.34133/bmr.0040 (PMC11205919; doi:10.34133/bmr.0040)
Supplement: Supplementary 1 — Figs. S1 to S3 [file bmr.0040.f1.docx]

**Enhancing Cancer Therapy: Boron-Rich Polyboronate Ester Micelles for Synergistic BNCT and PD-1/PD-L1 Checkpoint Blockade**

Yi-Lin Chiu, Wan Yun Fu, Wei-Yuan Huang, Fang-Tzu Hsu, Hsin-Wei Chen, Tzu-Wei Wang, **Pei Yuin Keng^*^**

Department of Material Science and Engineering, National Tsing Hua University, Hsinchu City 300, Taiwan

Correspondence: [keng.py@mx.nthu.edu.tw](mailto:keng.py@mx.nthu.edu.tw)

Supporting information


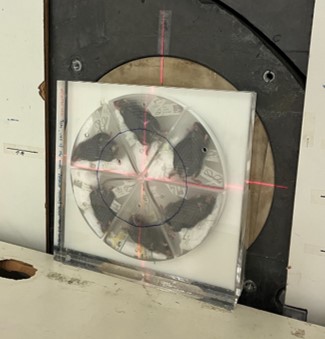


**Figure S1.** The acrylic holder (transparent) was used to fix the mice using paper tape during the BNCT neutron irradiation and covered with polyethylene (PE) board (white), and positioned the right hindlimb towards the center of the holder. The holder was placed at the center of the neutron irradiation tunnel.


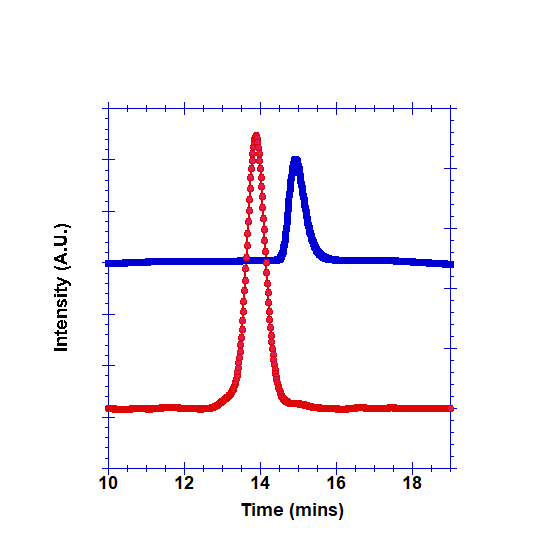


**Figure S2** Overlay GPC curves of the mPEG-b-(PVB-r-PVBE) (red trace) amphiphilic block copolymer and the mPEG-Br macroinitiator (blue trace).
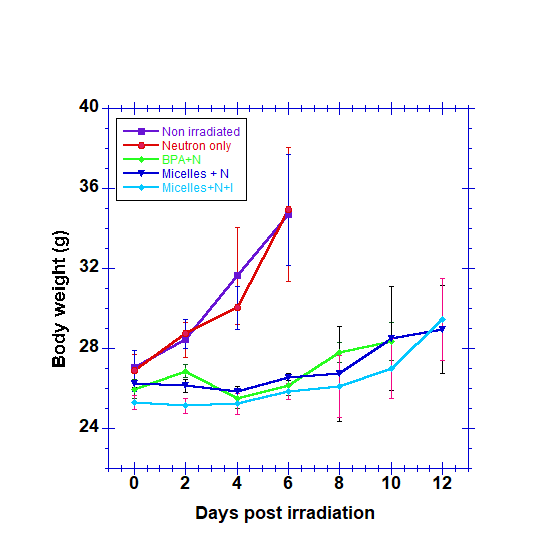


**Figure S3:** The mouse body weight after BNCT and mice in the control group.
